# PLS-DA Model for the Evaluation of Attention Deficit and Hyperactivity Disorder in Children and Adolescents through Blood Serum FTIR Spectra

**DOI:** 10.3390/molecules26113400

**Published:** 2021-06-03

**Authors:** Gulce Ogruc Ildiz, Ahmet Karadag, Ersin Kaygisiz, Rui Fausto

**Affiliations:** 1 Department of Physics, Faculty of Sciences and Letters, Istanbul Kultur University, 34158 Istanbul, Turkey; karadagahmet23@gmail.com; 2 Department of Chemistry, CQC, University of Coimbra, P-3004-535 Coimbra, Portugal; rfausto@ci.uc.pt; 3 Department of Geological Engineering, Istanbul University-Cerrahpasa, 34320 Istanbul, Turkey; ersinkygsz@gmail.com

**Keywords:** attention deficit and hyperactivity disorder (ADHD), FTIR spectroscopy, chemometrics, PLS-DA

## Abstract

Attention deficit and hyperactivity disorder (ADHD) is one of the most common neurodevelopmental disorders of childhood. It affects ~10% of the world’s population of children, and about 30–50% of those diagnosed in childhood continue to show ADHD symptoms later, with 2–5% of adults having the condition. Current diagnosis of ADHD is based on the clinical evaluation of the patient, and on interviews performed by clinicians with parents and teachers of the children, which, together with the fact that it shares common symptoms and frequent comorbidities with other neurodevelopmental disorders, makes the accurate and timely diagnosis of the disorder a difficult task. Despite the large effort to identify reliable biomarkers that can be used in a clinical environment to support clinical diagnosis, this goal has never been achieved hitherto. In the present study, infrared spectroscopy was used together with multivariate statistical methods (hierarchical clustering and partial least-squares discriminant analysis) to develop a model based on the spectra of blood serum samples that is able to distinguish ADHD patients from healthy individuals. The developed model used an approach where the whole infrared spectrum (in the 3700–900 cm^−1^ range) was taken as a holistic imprint of the biochemical blood serum environment (spectroscopic biomarker), overcoming the need for the search of any particular chemical substance associated with the disorder (molecular biomarker). The developed model is based on a sensitive and reliable technique, which is cheap and fast, thus appearing promising to use as a complementary diagnostic tool in the clinical environment.

## 1. Introduction

Attention deficit and hyperactivity disorder (ADHD) is one of the most common neurodevelopmental disorders of childhood. In general it starts at an early age and may persist throughout the adult life.

ADHD is characterized by a series of symptoms, such as difficulty in sustaining attention, focusing and completing tasks, impulsive behavior and excessive activity [1,2,3]. According to the diversity of the symptoms, ADHD is classified into three main types [4,5,6]: predominantly inattentive, predominantly hyperactive-impulsive and the combined type. In the first presentation, individuals find it difficult to organize or finish a task, to pay attention to details and to follow instructions or dialogues, being easily distracted and frequently forgetting details of daily routines. In the second, the person fidgets and talks a lot, expressing difficulty to sit for a long duration to perform an activity, and has problems with control impulsivity. Younger children may run, jump or climb constantly and usually have more accidents and injuries than others. Although ADHD causes trouble in relationships and difficulties in school life for children and adolescents, no cure has been developed yet for the disorder, and its treatment usually involves the combination of medications, psychotherapy, counseling and lifestyle changes [7].

ADHD is estimated to affect ~10% of the world’s population of children, with rates that are similar between countries. It is diagnosed approximately twice more often in boys than in girls, though it is frequently unnoticed in girls because their symptoms are, in general, less disruptive. Interestingly, it does not appear to be related to the style of parenting or discipline. About 30–50% of those diagnosed in childhood continue to have characteristic ADHD symptoms later, and 2–5% of adults have the condition [8,9,10].

Many studies pointed to genetic, environmental and social factors as possible causes of ADHD, but its precise underlying etiology remains uncertain and appears to vary from case to case [1,2,3,11,12]. Current diagnosis of ADHD follows the Diagnostic and Statistical Manual of Mental Disorders (Fifth Edition; DSM-5) diagnosis criteria [4] and is based on the clinical evaluation of the patient, including interviews performed by clinicians with parents and teachers of the children, which makes it relatively subjective. The fact that ADHD shares common symptoms and frequent comorbidities with other neurodevelopmental disorders (e.g., autism spectrum disorder, bipolar disorder) [13,14] also contributes to making an accurate and timely diagnosis of the disorder a difficult task. However, early diagnosis facilitates the proper treatment of the symptoms and may considerably improve the life conditions of the young individuals suffering with ADHD.

A large number of studies using several different approaches (e.g., neuroimaging, metabolic and genetic investigations) have been carried out with relative success, in order to find the underlying causes of ADHD and correlate them with specific biomarkers. Unfortunately, so far, none of those approaches has been able to find reliable biomarkers that can be used in a clinical environment to support a clinical diagnosis [15,16,17,18,19,20,21,22,23,24,25]. Schmidt et al. [26] defined a biomarker as a characteristic that can be objectively measured and evaluated as an indicator of a normal biological process, a pathogenic process or a response to a therapeutic intervention. Most biomarker studies compare case patients and control subjects to determine the sensitivity and specificity of the biomarker for detecting the disorder. As stressed by Scassellati and coworkers [15], the mechanistic status of the biomarkers is typically not elucidated by such studies, as the biomarkers can be a measure of vulnerability to the disorder, of processes taking place with the onset of the disorder or leading to chronicity or to epiphenomena of the disorder. Biomarkers can also reflect effects of treatment or physiological responses to the stress of living with a chronic disorder. Nevertheless, the identification of appropriate biomarkers may still provide important clues to the understanding of the causes and mechanisms of the action of the diseases, stimulating studies in this area. In addition, the identification of peripheral biochemical markers, measurable with reliable, fast and non-invasive methods, may help the diagnosis of a given disorder in the clinical environment.

Body fluids are easily accessible, and their use for medical diagnostics is a common practice. The analysis of body fluids using vibrational spectroscopy (either Raman or infrared) has progressively gained the respect of clinicians as a complementary diagnostic tool, with advantages over other techniques, such as being a sensitive and reliable approach, but also cheap, fast and easily adaptable to the clinical environment [27,28]. When used together with modern chemometric methods, Raman and infrared spectroscopies are powerful analytical instruments, which can efficiently probe the biochemical environment of a given biological sample [27,28,29,30,31,32,33,34,35,36,37,38,39]. As they are very sensitive to molecular structure and intermolecular interactions, Raman and infrared spectra provide signatures of a biological sample, such as imprints of its molecular constitution and chemical environment. An additional advantage offered by this approach is that one does not need to look for spectroscopic evidence of a specific molecular biomarker, rather the whole spectrum of the sample represents a holistic view of its biochemical environment, i.e., the full spectral data may be used as a biomarker without the need to search for any particular chemical substance. Such a holistic approach does not allow for the extraction of information on the specific metabolic mechanisms associated with the disease or the precise chemical species involved. However, it is more reliable for the general characterization of the samples and for their differentiation from the controls since no information is ignored and the whole biochemical environments are subjected to scrutiny.

Recently, this approach has been successfully applied to the development of analytical models for evaluation of neurodevelopmental diseases, using principal component analysis (PCA) or partial least-squares discriminant analysis (PLS-DA) applied to Raman infrared spectroscopic data obtained from blood samples [27,29,30]. Autism spectrum disorder, bipolar disorder (including identification of its different characteristic phases) and schizophrenia are recent cases of the successful application of this strategy to neurodevelopmental disorders [27,29,30].

In the present study, infrared spectroscopy was used together with hierarchical clustering analysis and PLS-DA to develop a model based on the spectra of blood serum samples that can distinguish ADHD patients from healthy individuals. The obtained results indicate that the used approach may receive application as a complementary diagnostic tool in the clinical environment.

## 2. Materials and Methods

### 2.1. Clinical Stage

Thirty children and adolescent outpatients with diagnosed ADHD according to DSM-5 [4] criteria were included in this study. The patients were chosen from individuals admitted to the Child and Adolescent Psychiatry Clinic of Pamukkale University (Denizli, Turkey) during a time period of 3 months, their ages being 6–14 years (23 boys and 7 girls). Individuals with other psychiatric disorders and those having a chronic medical comorbid condition were excluded. Subjects were chosen from the patients that were not under medication. The blood samples of the controls were obtained during the same period of time as the ADHD patients, with the subjects being chosen to match the patients’ cohort regarding sex and age. The control group consisted of 29 healthy children and adolescents, 22 boys and 7 girls, with ages between 6 and 14 years, who did not have any psychiatric history and were not on medication. All parents approved their children’s participation in this study by giving written consent. The study was approved by the Ethics Committee of the Pamukkale University, Faculty of Medicine (date: 12 May 2015).

Five milliliters of blood were collected from each participant and placed in Vacutainer plastic SST (Serum Separator Tube) gel tubes in order to separate their serum from cellular material. The gel in the tubes creates a physical barrier between the serum and blood cells after centrifugation and accelerates the serum coagulation by means of silica particles on the tube wall. Without shaking, the tubes were gently inverted 4–5 times so that the blood made good contact with the silica particles. After 30 min, the blood coagulated spontaneously. Following clotting, the samples were centrifuged at 10,000 rpm for 15 min to separate the serum from the cellular material. The obtained serum samples were placed in Eppendorf tubes and frozen at −20 °C. This protocol was applied for all samples.

### 2.2. Spectroscopic Stage

One microliter of an unfrozen blood sample was placed on the attenuated total reflectance (ATR) unit and allowed to air-dry at room temperature (~10 min). ATR Fourier transform infrared (FTIR) spectra, within the 400–4000 cm^−1^ spectral range, were recorded with 4 cm^−1^ spectral resolution on a Perkin Elmer Spectrum One spectrometer, equipped with a KBr beam splitter and a deuterated triglycine sulfate (DTGS) detector, combined with a diamond Gladi ATR accessory (Pike Technologies). The samples for both ADHD and controls were ordered randomly for spectra recording. For each blood serum sample, 5 spectra (replicates) were obtained, each one being the average of 64 scans. Background was collected immediately prior to each sample measurement.

For the statistical analysis, the 3700–900 cm^−1^ spectral range was chosen. The spectra were processed by baseline correction using a linear function and area normalization. For outliers’ deletion, all spectra of each group (Control group (C) and ADHD group (A)) were subjected to principal component analysis (PCA) [40,41,42,43], using the Nonlinear Iterative Partial Least-Squares (NIPALS) algorithm [44], which allowed facile detection of the outliers by simple inspection of the score plots. After the exclusion of outliers (1 sample of the Control group), the average spectra of each sample and of each group were obtained. All data pre-processing was undertaken with the Unscrambler^TM^ CAMO software (Version 10.5) [45].

### 2.3. Statistical Stage

As an *a priori* test, the overall similarity of the samples within each group and the dissimilarity between the two groups were investigated by applying unsupervised hierarchical clustering analysis to all samples (30 ADHD, 28 Control) using the Ward’s algorithm with squared Euclidean distances [46,47].

For the development of the classification model, 20 samples belonging to the ADHD group and 18 samples belonging to the Control group were randomly chosen as the calibration set. To test the classification model (prediction), the remaining 10 samples from each group were used as the test set.

The classification model was established by using the PLS-DA method [48,49,50,51], with internal full cross-validation being applied during calibration [52]. The PLS-DA method consists in a classical PLS regression [50], but in this case the response variable *Y* is a categorical one, expressing the class membership of the samples. PLS-DA then encompasses two main procedures: PLS component construction (i.e., dimension reduction) and prediction model construction (discriminant analysis). A few underlying or latent factors related to the response (*Y*) and the observable (*X*) variables accounting for most of their variances can be found through this method. In practical terms, the latent factors played a similar role as the principal components in PCA, but a better discrimination between the different classes can, in general, be achieved because in PLS, the directions that are associated with high variation in the data are sought in the factor space, while the search is biased toward directions leading to accurate class prediction for the samples in the training set.

Interestingly, while formally in PLS-DA the response matrix is a column vector (strictly speaking, in PLS1-DA [51]) and is categorical (for a two classes experiment, *Y* values can be simply 0 and 1), it is internally recoded via an indicator variable, which allows the PLS regression to run as if *Y* is a continuous variable. This PLS classification trick works well in practice, as demonstrated by the success of the method.

The linear regression is expressed by Equation (1), where *B* is the regression coefficients vector to be determined, and *F* is the residuals vector, which is to be minimized (in fact, the sum of the squares of the residuals, $\overset{N}{\underset{i=1}{\sum }}f^{2}=F^{T}F$, is the effective quantity to minimize):

(1) $$Y=\overset{=}{X}B+F$$

(2) $$B={\left({\overset{=}{X}}^{T}\overset{=}{X}\right)}^{-1}{\overset{=}{X}}^{T}Y$$

The coefficients *B* can be obtained from Equation (2) if the matrix $\overset{=}{X}$ is full ranked, i.e., if its columns are linearly independent. However, this least-squares solution is ill-conditioned if the data matrix does not have full rank, which happens most often when the number of variables *p* exceeds the number of samples *N*. The solution is to project each measurement into a lower-dimensional subspace spanned by the data, which corresponds to define a reduced number of *k* latent variables (factors), each being a linear combination of the original set of variables. In practice, this is achieved by decomposing $\overset{=}{X}$ and *Y* as in Equations (3) and (4), and then solving Equation (5), where $\overset{=}{W}$ is a weight matrix, which during the regression is iteratively determined, being initialized as ${\overset{=}{X}}^{T}Y$.

(3) $$\overset{=}{X}=\overset{=}{U}{\overset{=}{V}}^{T}+\overset{=}{E}$$

(4) $$Y=\overset{=}{P}Q+F$$

(5) $$B=\overset{=}{W}{\left({\overset{=}{V}}^{T}\overset{=}{W}\right)}^{-1}Q^{T}$$

In Equations (3–5), $\overset{=}{U}$ and $\overset{=}{P}$ are the *X* and *Y* score matrices and $\overset{=}{V}$ and *Q* the corresponding *X* and *Y* loadings. $\overset{=}{E}$ and *F* are the residuals in *X* and *Y*, respectively.

Once the coefficients of the regression are obtained, they can be used for subsequent classification (prediction) purposes. First, the unknown samples are reduced into the new low-dimensional space (the one defined by the *k* PLS latent factors) using *B*, to produce the predicted values (*y*_pred_). Given a set of training data that contains *G* classes, the PLS-DA model produces *G* predicted values ($y_{pred}^{\left(1\right)}$, $y_{pred}^{\left(2\right)}$, …$y_{pred}^{\left(G\right)}$) for each sample to classify. Ideally, the perfect class membership should be ‘1′ or ‘0′ to indicate that the sample belongs to that class or not. However, as noted above, in practice the resulting predicted values are between 0 and 1, instead of an integer. For that reason, a decision rule (DR) has to be applied in order to translate the predicted value into a meaningful class membership. Many different decision rules can be used, and an interesting discussion on this subject can be found in Reference [51]. A commonly used DR classifies a sample as belonging to class *A* if the predicted value for that class $y_{pred}^{\left(A\right)}$ differs from the *Y* (integer) value of that class by less than ±*Y*/2. For example, using this DR, for a two-classes model with *Y* = 1, 0 (belong, not-belong) values for both classes, classification of the sample in a given class requires that the predicted *y*_pred_ value for that class stays in the range 1 ± 0.5.

All chemometric analyses were accomplished using the Unscrambler^TM^ CAMO software (Version 10.5) [45]. The prediction performance of the model was evaluated by calculating its sensitivity, specificity, precision, accuracy and efficiency statistical parameters [53,54].

## 3. Results and Discussion

### 3.1. Preliminary Data Analysis

The pre-processed data (as described above) were initially inspected using the heat map, average spectra difference profile and hierarchical cluster analysis.

**Figure 1:**
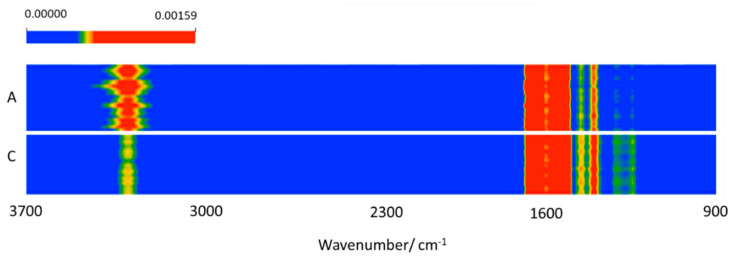
Heat map for the samples belonging to the two groups (**A**, ADHD; **C**, control).

**Figure 2:**
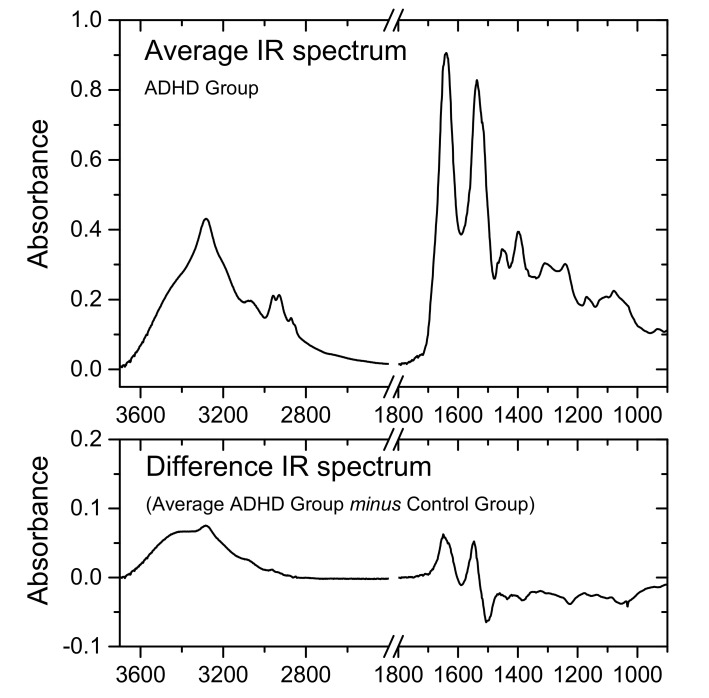
Average IR spectrum of ADHD group blood serum samples (3700–2400 and 1800–900 cm^−1^ regions; top panel), and difference spectrum obtained by subtracting the average spectrum of the Control group to the average spectrum of the ADHD group (bottom). The average IR spectrum of the Control group is provided in Figure S1 (Supplementary Materials).

The heat map is a graphical method for visualizing attribute values by class in a two-way matrix [55]. The values (IR intensities) are represented by colors, the *X* and *Y* axes relating to variables and samples, respectively. The latter were grouped according to their class (A or C) membership. The heat map (Figure 1) revealed distinct patterns for the spectra of ADHD patients compared to those of the control healthy individuals, in both the high- and low-frequency regions. By comparing the heat map with the average IR spectrum the blood serum of the ADHD Group and the difference spectrum obtained by subtracting the average spectrum of the Control Group to the average spectrum of the ADHD Group, which are shown in Figure 2, it can be concluded that the data seems to indicate that the blood serum of the ADHD patients has an increase of protein total contents (as shown by the higher relative intensity of the protein characteristic amide A, I and II bands in the ranges 3640–3100, 1700–1590 and 1590–1480 cm^–1^, respectively) [38,39,56,57,58] and a slight decrease of tyrosine (as shown by the lower intensity in the region of the tyrosine characteristic bands at ~1350 and 1250 cm^–1^ bands) [59] compared to the control group. These results shall be considered only as indicative; however, a deficiency in the trace amine phenylethylamine, from which tyrosine is a precursor, was reported for ADHD patients [15,60,61]. This result, as well as the increased level of the total protein contents in the blood serum of children with ADHD, is also in consonance with similar observations for children affected by other neurodevelopmental disorders, such as autism [27,29,62,63,64].

**Figure 3:**
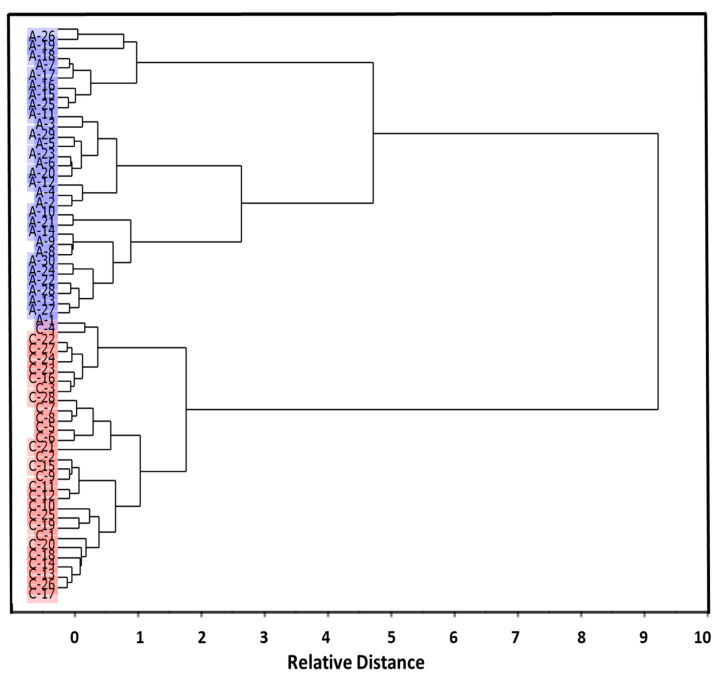
Hierarchical cluster analysis of ADHD (A; highlighted in blue) and control (C; highlighted in red) groups’ blood serum IR spectra, according to the Ward’s method, using squared Euclidean distances.

The dendrogram obtained from the hierarchical clustering analysis conducted on all samples (Figure 3) showed a clear discrimination of the samples belonging to the ADHD group from the Control group. It is worth mentioning that the samples belonging to the Control group appeared as more homogenous in comparison to those belonging to the ADHD group. This could be expected, taking into account the illness variability, which may result in a range of slightly different blood serum biochemistry.

### 3.2. Development of Classification Model

A classification model was built using the PLS-DA method, as explained in Section 2.3. The model was developed using five latent variables (Factors), but it was seen that the first three Factors accounted for over 98% of the variance in both *X* and *Y* variables. The root-mean-square errors (RMSEs) for training and validation were calculated as 0.08 and 0.10, respectively, indicating the excellent quality of the regression.

**Figure 4:**
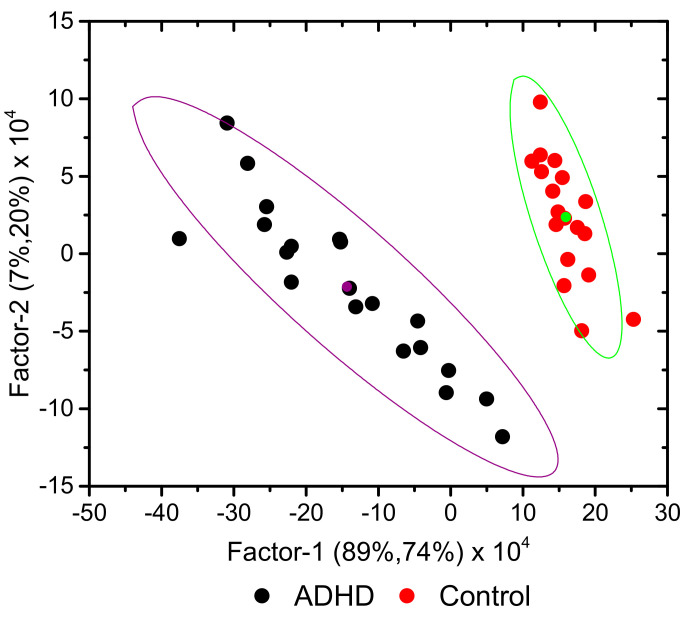
A 2D scores plot (Factor-1 vs. Factor-2) for the developed PLS-DA model, showing the 95% confidence ellipses. The points with the same color of the ellipses correspond to their centers and are the average point of the associated distribution.

Figure 4 shows the 2D (Factor-2 vs. Factor-1) scores plot for the developed model (including the 95% confidence ellipses), where a clear discrimination between the ADHD and Control groups can be observed along Factor-1. Factor-1 explained 89 and 74% of the total variance in the *X* and *Y* variables, respectively, in the training set (Factor-2 and Factor-3 explained 7 and 2% variance in *X*, and 20 and 4% variance in *Y*, respectively), the numbers being identical for validation. As it could be anticipated, the loadings for the discriminative Factor-1 (see Figure S2, in the Supplementary Materials) were found to essentially reproduce the difference IR spectrum obtained by subtracting the average spectrum of the Control Group from the average spectrum of the ADHD Group shown in Figure 2.

Similarly to what was seen in the cluster analysis dendrogram (Figure 3), and by the same reasons, the samples belonging to ADHD Group appeared more dispersed in the PLS-DA scores plots than those belonging to the Control Group.

### 3.3. Predictions

The prediction accuracy of the developed PLS-DA model was tested using 10 samples from each group (ADHD and Control groups) that were kept out of the calibration set. All spectra belonging to the test set were pre-processed using the same methodology used for calibration set samples.

**Figure 5:**
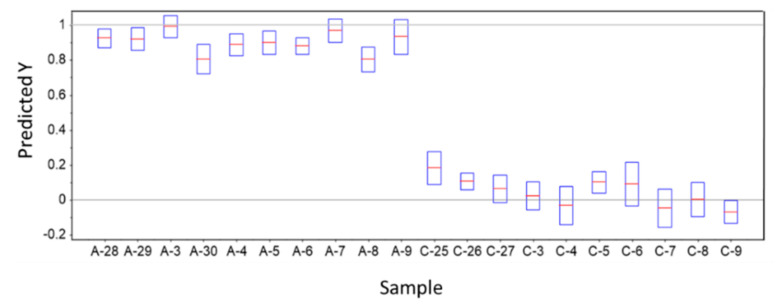
PLS-DA model predicted *Y* values for ADHD (A) and Control (C) test samples. The predicted values are indicated by the horizontal red lines and the deviations by the blue boxes. In the model, samples belonging to the Control Group define a class with reference *Y* value equal to 0, and those belonging to ADHD patients define a class with reference *Y* value equal to 1.

**Figure 6:**
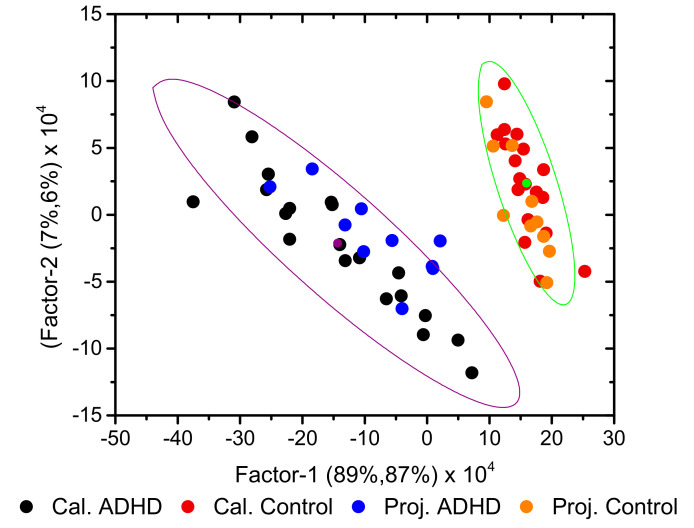
2D scores plot (Factor-2 vs. Factor-1) of the developed PLS-DA model, showing the calibration (Cal.) and projected test set (Proj.) samples. The 95% confidence ellipses for the calibration set are shown. The points with the same color of the ellipses correspond to their centers and are the average point of the associated distribution.

The results of the model predictions are summarized in Figure 5 and Figure 6. The criterion used to classify the samples as belonging to a given group was that the corresponding predicted *Y* value falls within ±0.5 relative to the corresponding *Y* reference values (0 for control samples, and 1 for ADHD samples). All predicted *Y* values for the tested samples were found to stay within the range of values established by the used classification criterion for assignment of the samples to their proper class (both for Control and ADHD tested samples; see Figure 5). In other words, the model was able to correctly classify all samples, corresponding to the superlative case where maximal values for the model performance statistical indicators (sensitivity, specificity, precision, accuracy and efficiency performance parameters [53,54]) were obtained.

The projections of the test samples on the 2D Factor-2 vs. Factor-1 scores plot of the model are shown in Figure 6, which provides a simple visual illustration of the classification ability of the model. As it is shown in the figure, with a single exception, all projected predicted points remained within the 95% confidence ellipses of the corresponding class for the calibrating set.

## 4. Conclusions

In this study, infrared spectroscopy and multivariate statistical methods (hierarchical clustering and partial least-squares discriminant analysis (PLS-DA)) were used to develop a prediction model based on the spectra of blood serum samples. The model was able to distinguish ADHD patients from healthy individuals with an accuracy of 100% (for the tested samples). The approach used to develop the model considered the whole infrared spectrum (in the 3700–900 cm^−1^ range) as a holistic imprint of the biochemical blood serum environment (spectroscopic biomarker), overcoming the need for the search of any particular chemical substance associated with the disorder (molecular biomarker). The model relied on a sensitive and reliable spectroscopic technique that is also cheap and fast, which facilitates its practical applications. Overall, the obtained results indicated that the applied approach is promising when used as a complementary diagnostic tool for ADHD in the clinical environment.

## Data Availability

Not Applicable.

## Supplementary Materials

The following are available online, Figure S1: Average IR spectrum of Control Group blood serum samples (3700–2400 and 1800–900 cm−1 regions. The average spectrum of the ADHD Group is also depicted (thin line) for comparison, Figure S2: Loadings of of Factor-1 and Factor-2 of the developed PLS-DA classification model. The loadings are plotted multiplied by the factor-10 for a better comparison between the loadings of Factor-1 and the difference spectrum between the average IR spectra of the ADHD and the Control groups shown in Figure 2 of the article.
