# Supplementary material for: PLS-DA Model for the Evaluation of Attention Deficit and Hyperactivity Disorder in Children and Adolescents through Blood Serum FTIR Spectra
Source: Molecules. 2021 Jun 3;26(11):3400. doi: 10.3390/molecules26113400 (PMC8199998; doi:10.3390/molecules26113400)
Supplement: Supplementary file 1 [file molecules-26-03400-s001.zip › molecules-1211833-supplementary.pdf]

Supporting Information for

PLS-DA Model for the Evaluation of Attention Deficit and Hyperactivity Disorder in Children and Adolescents through Blood Serum FTIR Spectra

Gulce Ogruc Ildiz,[1,2,*] Ahmet Karadag,[1] Ersin Kaygisiz[3] and Rui Fausto[2]

[1] Department of Physics, Faculty of Sciences and Letters, Istanbul Kultur University, 34158 Istanbul, Turkey

[2] Department of Chemistry, CQC, University of Coimbra, P-3004-535 Coimbra, Portugal

[3] Department of Geological Engineering, Istanbul University-Cerrahpasa, 34320 Istanbul, Turkey

* Correspondence: g.ogruc@iku.edu.tr ; Tel.: +90 (212) 498 4319

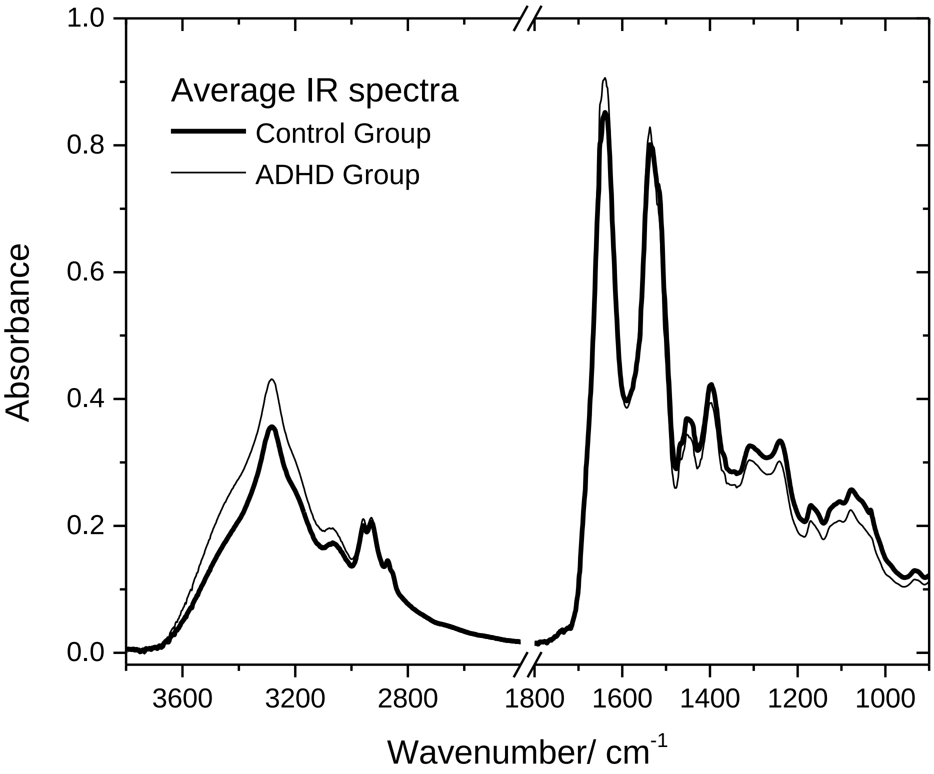

Figure S1. Average IR spectrum of Control Group blood serum samples (3700–2400 and 1800–900 cm^{-1} regions. The average spectrum of the ADHD Group is also depicted (thin line) for comparison.

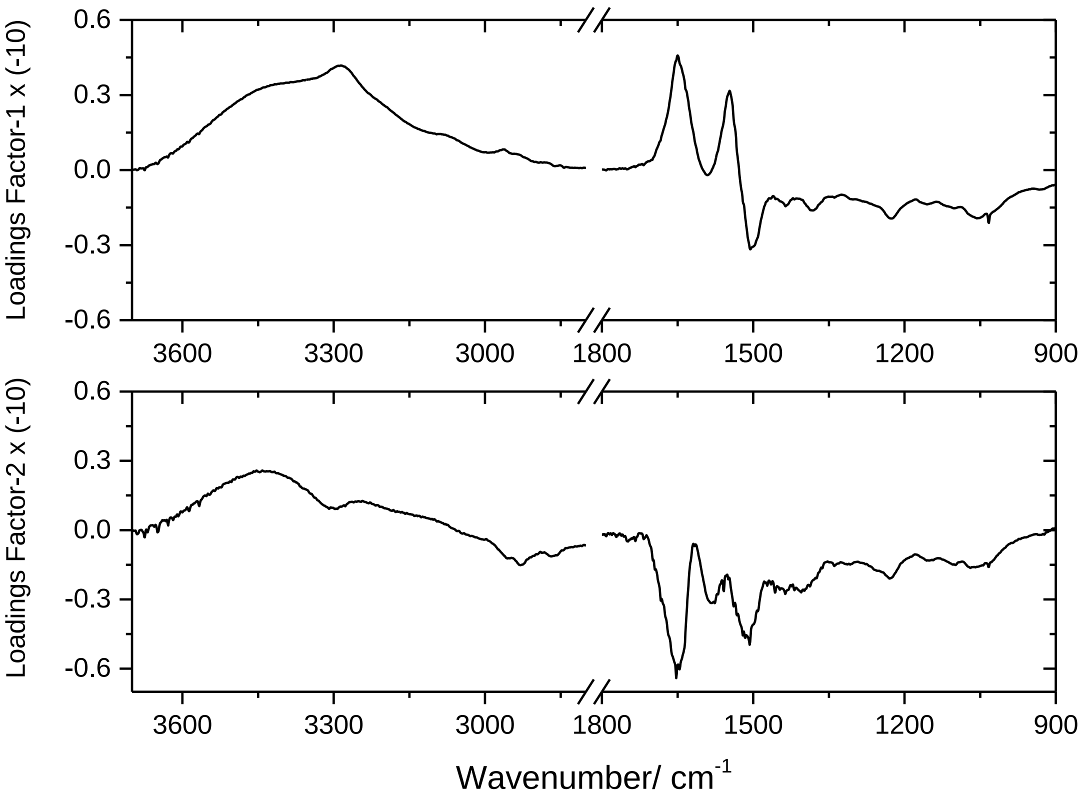

Figure S2. Loadings of of Factor-1 and Factor-2 of the developed PLS-DA classification model. The loadings are plotted multiplied by the factor -10 for a better comparison between the loadings of Factor-1 and the difference spectrum between the average IR spectra of the ADHD and the Control groups shown in Figure 2 of the article.
